# PSMB5 overexpression is correlated with tumor proliferation and poor prognosis in hepatocellular carcinoma

**DOI:** 10.1002/2211-5463.13479

**Published:** 2022-09-22

**Authors:** Jun Liu, Jinglin Mi, Shiqian Liu, Haiqiang Chen, Li Jiang

**Affiliations:** ^1^ Guangxi Medical University Nanning China; ^2^ Department of Radiation Oncology The First Affiliated Hospital of Guangxi Medical University Nanning China

**Keywords:** diagnosis, prognosis, proliferation and hepatocellular carcinoma, PSMB5

## Abstract

Aberrant expression of members of the proteasome subunit beta (PSMB) family (including PSMB2, PSMB4, PSMB7 and PSMB8) has been reported in hepatocellular carcinoma (HCC). However the role of PSMB5 in HCC is unclear. To address this issue, we examined the expression of PSMB5 in HCC tissues using the The Cancer Genome Atlas, International Cancer Genome Consortium and Gene Expression Omnibus databases. A quantitative real‐time PCR and immunohistochemistry were performed to validate the expression of PSMB5 in HCC. The survival mutation status and immune cell infiltration of PSMB5 were also evaluated in HCC. We then examined the effect of knocking down PSMB5 expression through RNA interference in the HCC cell line Huh7. High expression of PSMB5 was observed in HCC tissues and was associated with poor prognosis. PSMB5 expression and clinical characteristics were then incorporated to build a prognostic nomogram. We observed that PSMB5 expression was closely related to the abundance of B cells, CD4^+^ T cells, CD8^+^ T cells, dendritic cell macrophages and neutrophils. Moreover silencing of PSMB5 in Huh7 significantly suppressed cell proliferation and migration at the same time as increasing apoptosis. Inhibition of the phosphatidylinositol‐3‐kinase/Akt/mechanistic target of rapamycin pathway was observed after PSMB5 downregulation in Huh7 cells. Our findings suggest that PSMB5 may promote the proliferation of HCC cells by inactivating the phosphatidylinositol‐3‐kinase/Akt/mechanistic target of rapamycin signaling pathway and thus PSMB5 may have potential as a biomarker for diagnosis and prognosis of HCC.

## Graphical abstract

Proteasome subunit beta (PSMB)5 plays a critical role in regulating the content of particular proteins in humans and in removing misfolded proteins. In the present study, we observed that PSMB5 is overexpressed in hepatocellular carcinoma (HCC) tissues and high levels of PSMB5 expression are associated with worse overall survival. Knockdown of PSMB5 expression reduced the proliferation and migration of HCC cells but increased their apoptosis. PSMB5 may have potential as a biomarker for diagnosis and prognosis of HCC.

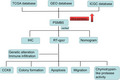

**Abbreviations**
AUC: area under the curve
CI: confidence interval
GEO: Gene Expression Omnibus
HCC: hepatocellular carcinoma
ICGC: International Cancer Genome Consortium
IHC: immunohistochemistry
LIHC: liver hepatocellular carcinoma
mTOR: mechanistic target of rapamycin
OS: overall survival
PI3K: phosphatidylinositol‐3‐kinase
PSMB: proteasome subunit beta
qRT‐PCR: quantitative real‐time PCR
ROC: receiver operating characteristic
siRNA: small interfering RNA
TCGA: The Cancer Genome Atlas
TNBC: triple‐negative breast cancer
UPP: ubiquitin‐proteasome pathway

According to the data collected in 2020 liver cancer is the sixth leading cause of malignant cancer and the third most prevalent cause of cancer death [1]. Hepatocellular carcinoma (HCC) is the most common type of predominant form liver cancer accounting for approximately 90% of all cases [1]. The main risk factors for HCC include hepatitis B virus or hepatitis C virus infection aflatoxin exposure, alcohol‐related cirrhosis, fatty liver disease, smoking and others [2].

Despite the significant advancement in the diagnosis and management of HCC, the efficacy of treatment and long‐term prognosis remain unsatisfactory, with the 5‐year overall survival of liver hepatocellular carcinoma (LIHC) being < 14.1% in China [3, 4, 5]. Thus, it is imperative to improve the prognosis by identifying the novel diagnostic biomarkers and therapeutic targets of LIHC.

The ubiquitin‐proteasome pathway (UPP) is a major pathway that degrades more than 80% of intracellular proteins in eukaryotes [6]. UPP is assembled by ubiquitin (Ub), ubiquitin activating enzyme (E1), ubiquitin binding enzyme (E2), ubiquitin protein ligase (E3) and 26S proteasomes [7]. On the other hand, the 26S proteasome is assembled by 20S proteasome and 19S regulatory particles, whereas PSMB5 is a core subunit of the 20S proteasome [8]. Evidence has suggested that PSMB5 is associated with antigen presentation and oxidative stress [9, 10]. In addition, the tumor‐promoting role of PSMB5 has been reported in relation to many human cancers, such as breast cancer, esophageal squamous cell carcinoma and prostate cancer. Aberrant expression of PSMB5 is correlated to tumor cell proliferation metastasis and drug resistance [7, 11, 12, 13, 14]. However, as for a subunit of the whole proteasome complex, the functional role and biological mechanism of PSMB5 in HCC still remain largely unknown.

In the present study, it was observed that PSMB5 was upregulated in HCC tissue based on analysis of some databases, including The Cancer Genome Atlas (TCGA), International Cancer Genome Consortium (ICGC) and Gene Expression Omnibus (GEO). In addition, the mRNA and protein expression levels of PSMB5 were further validated by a quantitative real‐time PCR (qRT‐PCR) and immunohistochemistry (IHC). Then the expression of PSMB5 was identified as a risk factor for prognosis through Kaplan–Meier analysis and univariate analysis with a nomogram mode integrating PSMB5 expression level and clinical characteristics constructed to predict the survival rate. The genetic alteration and immune cells infiltration of PSMB5 were also analyzed. Furthermore, the underlying molecular functions and relevant pathway of PSMB5 were investigated.

## Materials and methods

### Data collection

The transcriptome profiles and clinical data information of cohorts included TCGA‐LIHC ICGC‐LIRI‐JP and GSE14520. The TCGA cohort contained 370 HCC tissues and 50 normal tissues, the GEO cohort contained 247 HCC tissues and 241 normal tissues, and the ICGC cohort contained 232 HCC tissues and 202 normal tissues. PSMB5 expression level was compared between HCC tissues and normal liver tissues. The diagnostic values of PSMB5 were analyzed using the diagnostic receiver operating characteristic (ROC) curves and all the area under curve (AUC) was calculated.

### Survival analysis

Based on the median expression level of PSMB5, all of the HCC patients in the TCGA and ICGC cohorts were divided into the high‐PSMB5 group and the low‐PSMB5 group. Then, survival probabilities were calculated by means of Kaplan–Meier analysis and a log‐rank test. The prognostic role of PSMB5 was further explored in Kaplan–Meier plotter online database (http://kmplot.com/analysis). All of the factors with a smaller *P*‐value than 0.05 in univariate Cox analysis were inputted into the stepwise multivariate Cox regression analysis. The prognostic factors with *P* > 0.05 based on the result of multivariate Cox regression analysis were included to establish the nomogram. Concordance index (C‐index) and calibration curve were applied to validate the predictive accuracy of the nomogram.

### Genetic alteration analysis

The cBioPortal database (www.cbioportal.org), an integrative resource, was applied to investigate alterations in the PSMB5 gene. We selected ‘TCGA PanCancer Atlas’ in the ‘Query’ module and entered ‘PSMB5’ for queries of the genetic alteration. Alteration frequency copy number alteration and mutation type data are displayed in the ‘Cancer Types Summary’ module. The mutated site date of PSMB5 in HCC was shown as a schematic diagram and the 3D (Three‐dimensional) structure was downloaded by clicking the ‘View 3D Structure’. Survival differences for HCC with or without PSMB5 genetic alteration were also presented by clicking the ‘Comparison’ module.

### Immune infiltration analysis

The TIMER database (https://cistrome.shinyapps.io/timer/) is a user‐friendly web tool that can be used to conduct systematic analysis of immune cells infiltration in diverse cancer from the TCGA database. The relationship between PSMB5 expression and six types of infiltrating immune cells (B cells, CD4^+^ T cells, CD8^+^ T cells, dendritic cell, macrophages and neutrophils) was estimated using TIMER algorithm, and tumor purity was also estimated. Moreover, the ‘correlation module’ in the TIMER and GEPIA databases (http://gepia.cancer‐pku.cn/) was applied to explore the correlation between PSMB5 expression and the gene markers of immune cells.

### Patient samples

Fifteen pairs of HCC and corresponding adjacent tissues were collected from The First Affiliated Hospital of Guangxi Medical University. All of these patients provided informed consent in writing for the study. This project was approved by The Ethics Committee of the First Affiliated Hospital of Guangxi Medical University and the approval number was 2021(KY‐E‐237). The study was conducted according to the Declaration of Helsinki.

### Cell line and transfection

Normal human liver cell line L02 and HCC cell lines SK‐hep1, SNU‐449, SMMC‐7721, HCC‐LM3, Huh7 and MHCC‐97H were obtained from American Type Culture Collection (Manassas, VA, USA) or Chinese Academy of Sciences Cell Bank (Shanghai, China). SK‐hep1, SNU‐449 and SMMC‐7721 were grown in RPMI‐1640 medium (Gibco, Waltham, MA, USA) plus 10% fetal bovine serum (Gibco), whereas other cells were grown in Dulbecco's modified Eagle's medium plus 10% FBS. 1% Penicillin and streptomycin were added into the medium. The cell line was cultured at 37 °C with 5% CO_2_ in a humidified incubator. PSMB5‐small interfering RNAs (siRNAs) and their corresponding negative control were synthesized by GenePharma (Shanghai, China). When the cell density reached the range 70–80%, the transfection was performed using Lipofectamine 3000 (Invitrogen, Carlsbad, CA, USA). The efficiency of the transfection was verified though qRT‐PCR analysis and western blotting.

### qRT‐PCR

The total RNA of cell and liver tissues was extracted with the assistance of TRIzol reagent (Takara, Shiga, Japan) and then reverse transcribed into cDNA using a cDNA synthesis kit (Thermo Fisher Scientific, Waltham, MA, USA), in accordance with the manufacturer's instructions. The cDNA was amplified using SYBR green mix (Bio‐Rad, Hercules, CA, USA) in a real‐time system (CFX96; Bio‐Rad). The relative mRNA expression level of PSMB5 was determined using the$2^{-\Delta \Delta C_{t}}$ method with ACTIN as an internal control. The specific primers are: ACTIN: 5′‐CCTGGCACCCAGCACAAT‐3′ (forward), 5′‐GGGCCGGACTCGTCATAC‐3′ (reverse); PSMB5: 5′‐GCTACAGCGGGTGCTTACAT‐3′ (forward), 5′‐TTCCCAGAAGCTGCAATCCG‐3′ (reverse).

### Western blotting

HCC cells were lysed using the RIPA buffer with protease inhibitors. The protein samples were separated by means of SDS/PAGE gels electrophoresis separation and then blotted onto poly(vinylidene difluoride) membranes. After 1 h of blocking with 5% skim milk, the membranes were incubated at 4 °C overnight with the corresponding primary antibodies against PSMB5 (Thermo Fisher), phosphatidylinositol‐3‐kinase (PI3K) (SAB, Baltimore, MD, USA), p‐PI3K (Affinit, JiangSu, China), Akt (Proteintech, Rosemont, IL, USA), P‐Akt (Proteintech), mechanistic target of rapamycin (mTOR) (Abcam, Cambridge, UK), p‐mTOR (Proteintech) and actin (Abcam). Then, secondary antibody (horseradish peroxidase‐conjugated) was applied to incubate membranes for 2 h at room temperature. ECL solution was also used to visualize the membranes. The protein bands on the membranes were obtained through the Typhoon fa9500 system (GE Healthcare, Chicago, IL, USA).

### Immunohistochemistry

HCC and adjacent tissues were fixed in 10% formalin for 12 h. The sections with a 5‐μm thickness were cut from the paraffin‐embedded specimens. After being deparaffinized, hydrated, and antigen retrieved, the sections were exposed to 3% H_2_O_2_ to quench the endogenous peroxidase activity, and were blocked by 5% goat serum. Then, the sections were incubated with the primary antibody of PSMB5 (Thermo Fisher) at 4 °C overnight. After washing in phosphate‐buffered saline, the sections were incubated with the secondary antibody for 1 h at 36 °C and the 3,3′‐diaminobenzidine method was adopted to detect the signal and hematoxylin was applied to reveal the nucleus. The immunoreactive score was calculated using the proportion of positive cells and the staining intensity. The proportion of positive cells was scored as: 0 (< 5%) 1 (5–25%), 2 (25–50%), 3 (50–75%) and 4 (> 75%). The staining intensity was scored as: 0 (negative), 1 (weak), 2 (moderate) and 3 (strong staining intensity). The final immunoreactive score was determined by summing the two scores.

### Cell proliferation assay

Cell proliferation was assessed using the CCK‐8 assay (Takara). In brief, 1000 cells per wells were seeded into a 96‐well plate and cultured for 0, 24, 48 and 72 h, respectively. CCK‐8 reagent was added and incubated in the incubator for 2 h. The absorbance value was determined at 450 nm with the assistance of a microplate reader.

### Colony formation assay

Colony formation was evaluated by means of a colony formation assay. Cells were inoculated in six‐well plates and incubated for 1 week. Then, the cells were washed using phosphate‐buffered saline and fixed using 4% paraformaldehyde for 15 min. After being stained with 1% crystal violet solution, the colonies were imaged and analyzed.

### Flow cytometry analysis

The apoptosis of HCC cell line was assessed through flow cytometry. 1.2 × 10^5^ cells per well were plated into six‐well plates. Flow cytometry analysis was performed after 48 h of culturing with the siRNA‐transfected cells. HCC cells were collected separately, and incubated with 7‐AAD and Annexin V‐APC (BioLegend, San Diego, CA, USA) in the dark for 15 min. Then, the stained cells were analyzed by means of flow cytometry.

### Cell migration

The cell migration ability of HCC cells was investigated using the transwell chamber system. In total, 5 × 10^4^ cells per well were seeded in the upper chamber with serum‐free culture medium and the medium containing 10% FBS was added into the lower chamber. After 48 h of incubation, invading and migrating cells were fixed with methanol, stained with 0.1% crystal violet and imaged using an inverted microscope. Three random fields were counted in each chamber.

#### Chymotrypsin‐like protease activity assay

The chymotrypsin‐like protease activity was measured using a Proteasome 20S Activity Assay Kit (Abcam). In brief, the cells were planted and added with proteasome assay loading solution. Then, the plate was incubated at room temperature for 2 h. The fluorescence intensity at an excitation/emission of 490/525 nm was detected using a fluorescence microplate reader.

### Statistical analysis

Statistical analysis and visualization were performed using r software, version 4.1.2 (R Foundation, Austria, Viennna) and associated packages, prism, version 8.0 (GraphPad Software Inc., San Diego, CA, USA) and spss, version 20.0 (IBM Corp., Armonk, NY, USA). The data across multiple groups were analyzed by one‐way analysis of variance, whereas those of two groups were analyzed by means of a *t*‐test. Chi‐squared tests were conducted to assess the correlation between PSMB5 expression and clinicopathological characteristics. *P* < 0.05 was considered significantly different.

## Results

### PSMB5 expression level in HCC

**Fig. 1:**
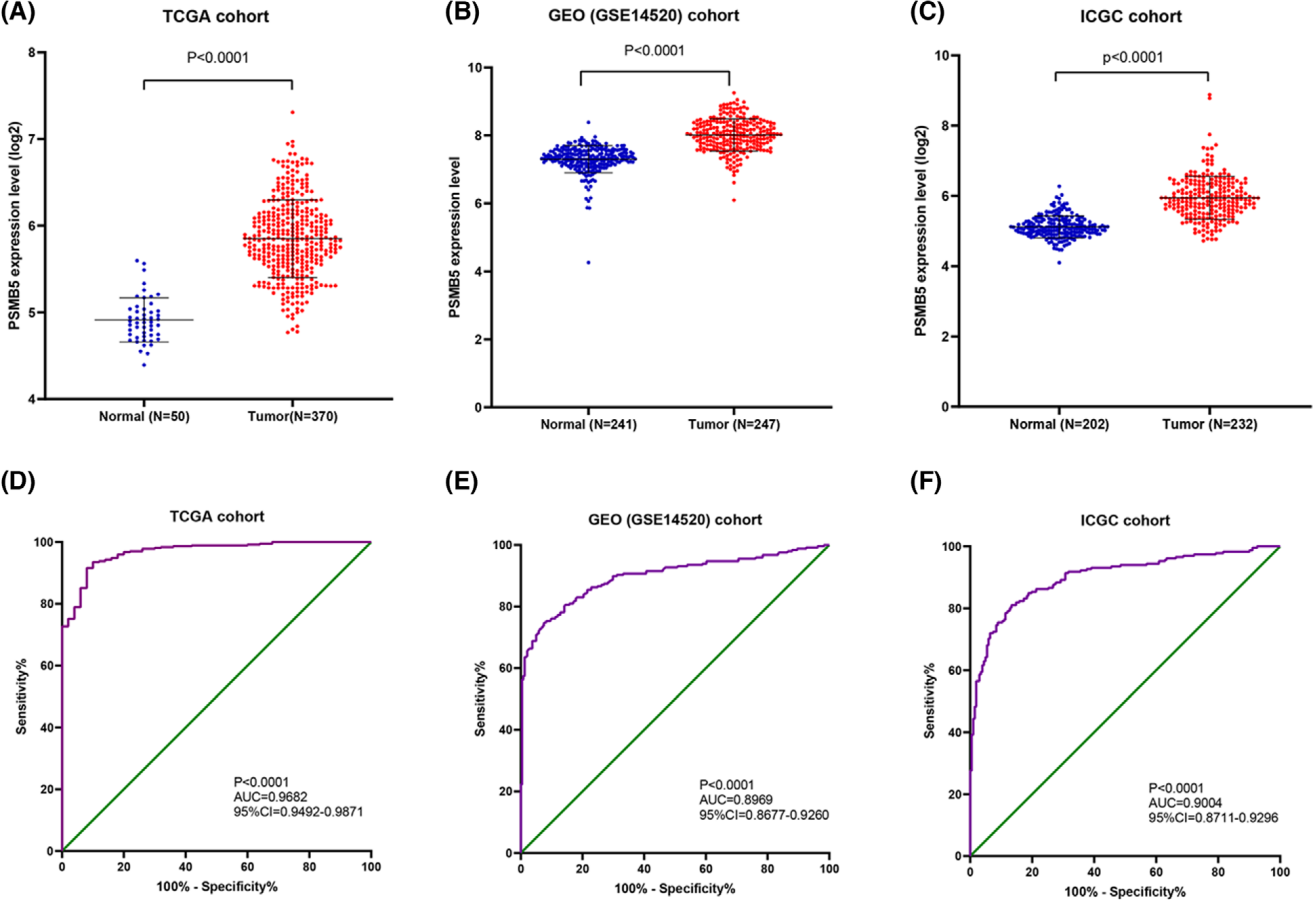
The expression of PSMB5 in HCC. (A–C) The expression of PSMB5 was signifcantly higher in HCC than normal tissue based on TCGA, GEO (GSE14520) and ICGC cohorts. (D–F) The diagnostic ROC curves of PSMB5 based on TCGA, GEO (GSE14520) and ICGC cohorts.

**Fig. 2:**
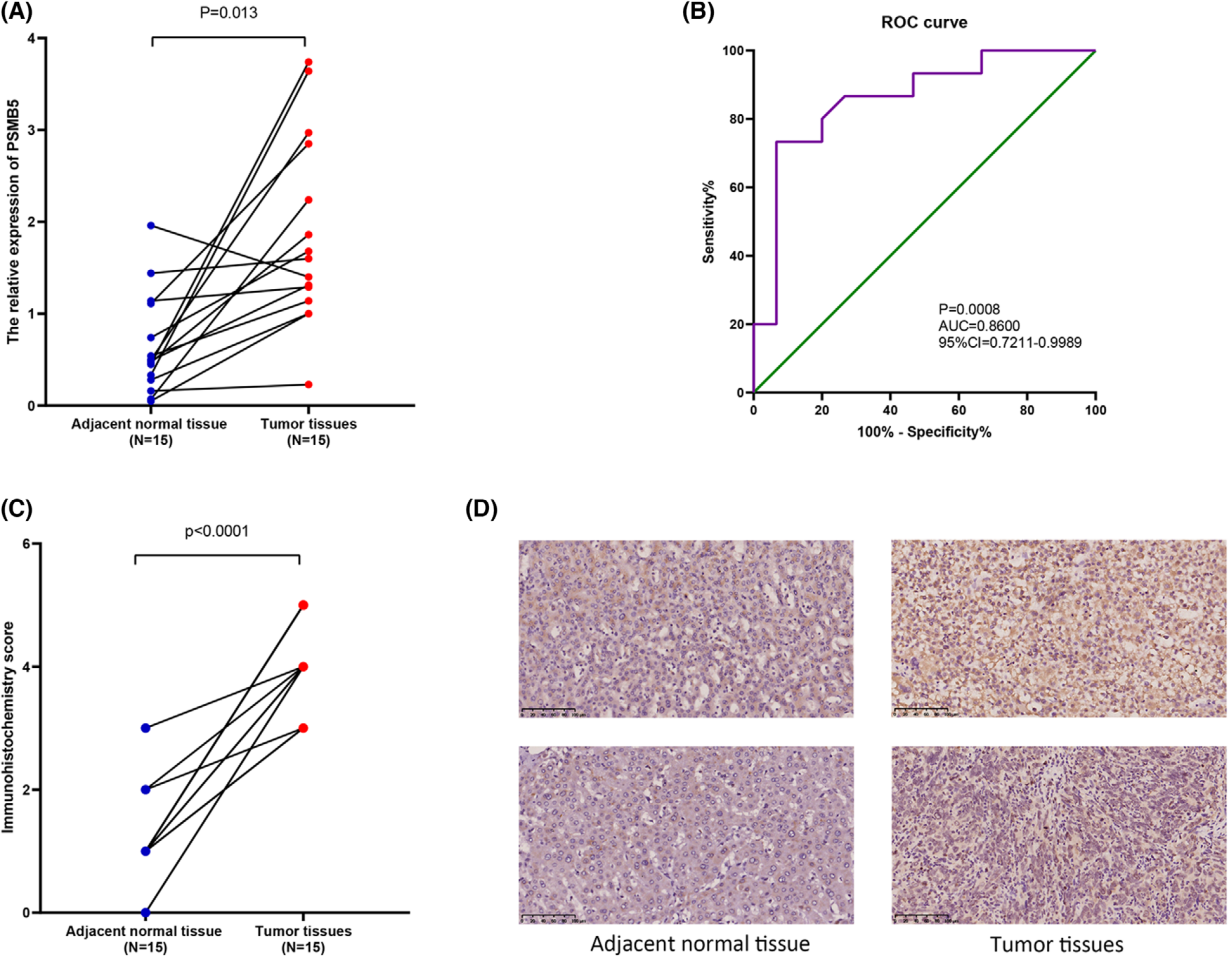
mRNA and protein expression of PSMB5 was confirmed by qRT‐PCR and immunohistochemistry. (A) mRNA expression level of PSMB5 between HCC and adjacent normal tissues. (B) The diagnostic ROC curves of PSMB5. (C, D) Immunohistochemistry score and protein expression of PSMB5 between HCC and adjacent normal tissues. Scale bars = 100 μm.

To investigate the expression of PSMB5 in HCC, a range of bioinformatics databases were analyzed. The result shows that the level of PSMB5 mRNA expression was significantly higher in the HCC tissues compared to the normal liver tissues derived from TCGA (*P* < 0.0001), GSE14520 (*P* < 0.0001) and ICGC (*P* < 0.0001) cohorts. As shown in the ROC curve, PSMB5 had high diagnostic value in TCGA [*P* < 0.0001, AUC (95% confidence interval [CI]) = 0.9682 (099492–0.9871)], GSE14520 [*P* < 0.0001, AUC (95% CI) = 0.8969 (0.9677–0.9260)] and ICGC [*P* < 0.0001, AUC (95% CI) = 0.9004 (0.8711–0.9296)] cohorts (Fig. 1). Then, a qRT‐PCR assay and IHC were conducted using 15 pairs of HCC and adjacent non‐tumor tissues. According to the result, PSMB5 mRNA (*P* = 0.013) and protein expression level (*P* < 0.0001) were higher in HCC tissues. ROC curves reveal that the PSMB5 had a diagnostic value in HCC (*P* = 0.0008, AUC = 0.8600, 95% CI = 0.7211–0.9989) (Fig. 2). The association between PSMB5 and the relevant genes is shown in Table S1.

### Clinicopathological parameters association and survival analysis of PSMB5

**Fig. 3:**
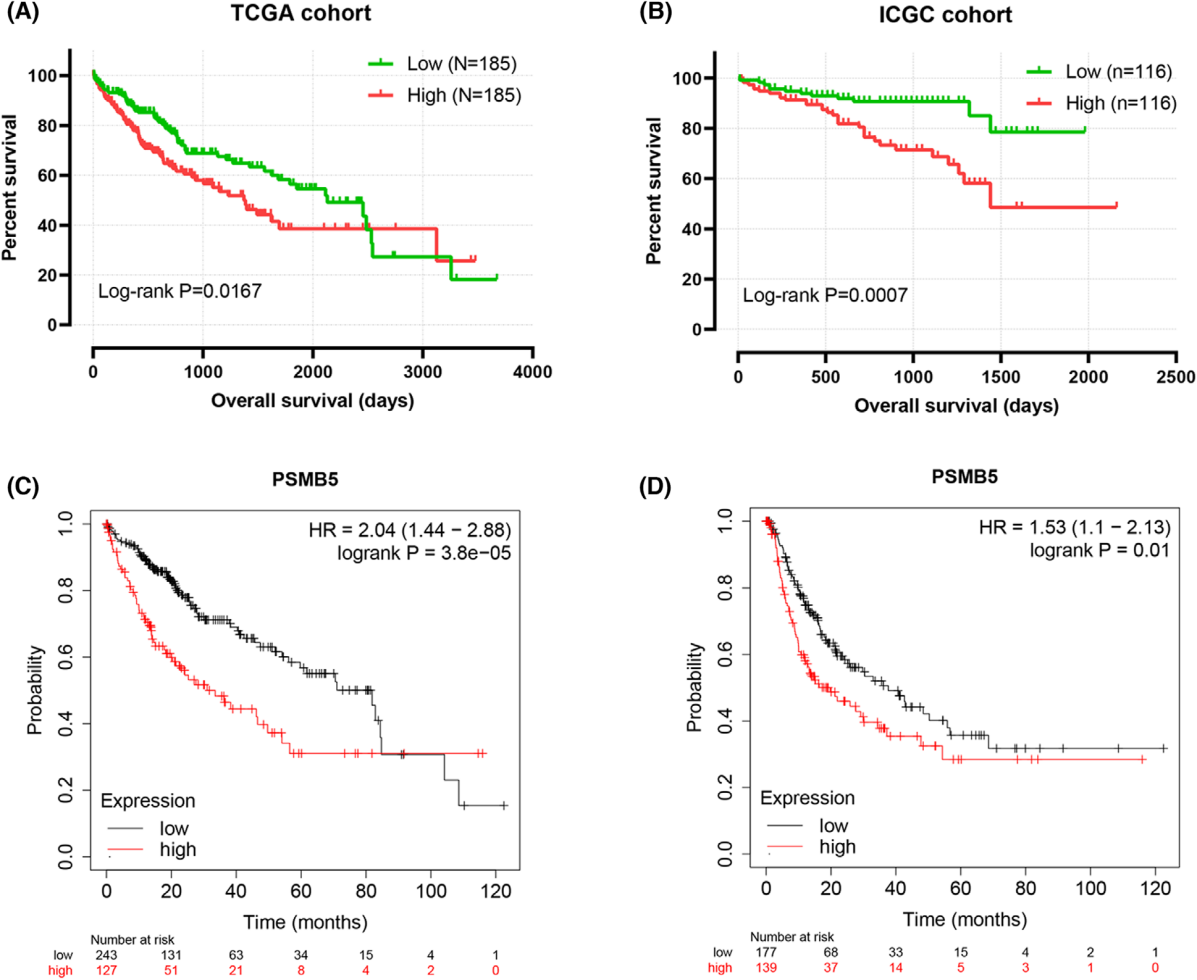
Kaplan–Meier survival curves for PSMB5 in HCC. (A) Overall survival curves of PSMB5 in HCC from the TCGA cohort. (B) Overall survival curves of PSMB5 in HCC from the ICGC cohort. (C, D) Overall survival and recurrence‐free survival curves of PSBM5 in HCC based on the Kaplan–Meier plotter database.

In total, 370 HCC samples in the TCGA cohort and 232 HCC samples in the ICGC cohort, as well as the specific patient information, were divided into two groups by the median expression of PSMB5. The distributions of clinical features indicated no significant difference between high and low PSMB5 expression groups (Tables 1 and 2). As indicated by the Kaplan–Meier curve, the high mRNA expression of PSMB5 was associated with the worse overall survival (OS) in the TCGA and ICGC cohorts (Fig. 3A,B). The Kaplan–Meier plotter online database was applied to further assess the prognostic value of PSMB5 in HCC, with the result showing that the HCC patients with a high level of PSMB5 expression had a worse OS and relapse free survival than those patients with a low level of expression (Fig. 3C,D).

**Table 1:** Clinical characteristics in TCGA cohort according to PSMB5 expression.

Characteristics	Low expression	High expression	*P* value
	(*n* = 185)	(*n* = 185)	
Age			
< 60 years	81	88	0.465
≧ 60 years	104	97	
Gender			
Female	62	59	0.74
Male	123	126	
T stage			
T1	100	81	0.15
T2	39	56	
T3	37	41	
T4	7	6	
Missing	2	1	
N stage			
N0	121	131	0.357
N1	3	1	
Missing	61	53	
M stage			
M0	135	131	0.369
M1	1	3	
Missing	49	51	
Clinical stage			
I	95	76	0.078
II	33	52	
III	41	44	
IV	2	3	
Missing	14	10	

**Table 2:** Clinical characteristics in ICGC cohort according to PSMB5 expression.

Characteristics	Low expression	High expression	*P* value
	(*n* = 116)	(*n* = 116)	
Age			
< 60 years	23	22	0.868
≧ 60 years	93	94	
Gender			
Female	29	32	0.655
Male	87	84	
Clinical stage			
I	24	12	0.081
II	51	55	
III	35	36	
IV	6	13	
Prior malignancy			
Yes	104	98	0.24
No	12	18	

### Predictive nomogram construction

**Fig. 4:**
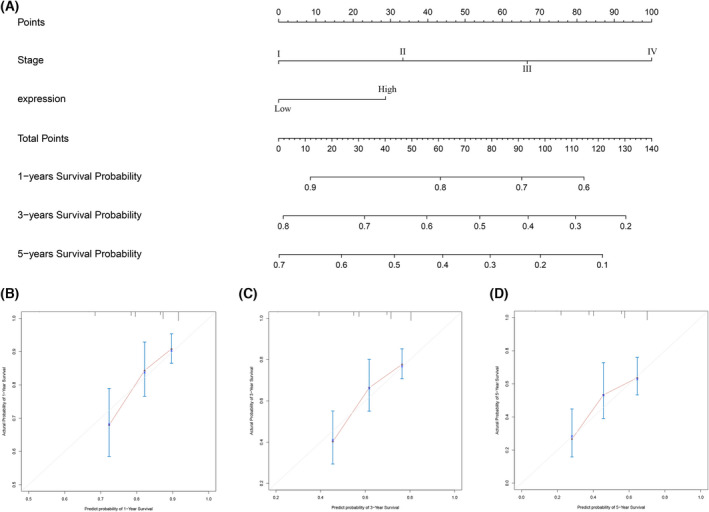
Construction of the nomogram consisted of PSMB5 expression and clinical features based on the TCGA cohort. (A) Nomogram. (B–D) Calibration curves of the nomogram to predict 1‐, 3‐ and 5‐year overall survival.

**Fig. 5:**
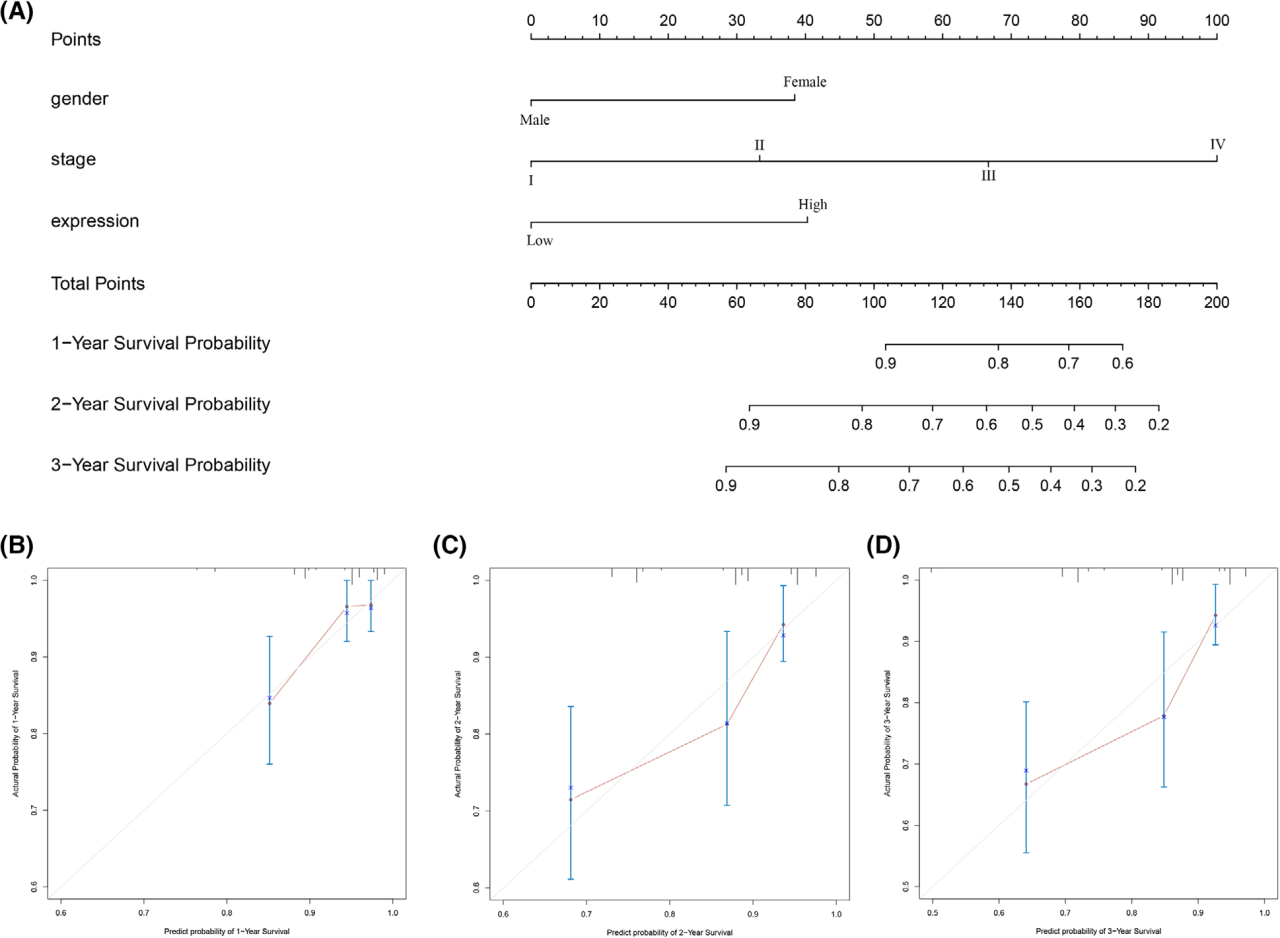
Construction of the nomogram consisted of PSMB5 expression and clinical features based on ICGC cohort. (A) Nomogram. (B–D) Calibration curves of the nomogram to predict 1‐, 2‐ and 3‐year overall survival.

Cox proportional hazard regression model was applied to identify the prognostic factors for LIHC patients. As shown in Tables 3 and 4, univariate analysis indicated that clinical stage and PSMB5 expression were significantly correlated with worse OS in the TCGA cohort, whereas gender, clinical stage and PSMB5 expression were significantly correlated with worse overall survival in the ICGC cohort. Multivariate analysis showed that clinical stage and PSMB5 expression were the independent risk factors for worse OS in the TCGA cohort, whereas gender, clinical stage and PSMB5 expression were the independent risk factors for worse OS in the ICGC cohort. Subsequently, nomogram was established for the prediction of 1‐, 2‐, 3‐ and 5‐year OS based on the result of multivariate analysis (Figs 4 and 5). Potential covariates involved clinical stage (stage I, 0 points; stage II, 33 points; stage III, 67 points; stage IV, 100 points) and PSMB5 expression (low expression, 0 points; high expression, 29 points) in the TCGA cohort, whereas potential covariates involved gender (female, 38 points; male, 0 points), stage (stage I, 0 points; stage II, 33 points; stage III, 67 points; stage IV, 100 points), PSMB5 expression (low expression, 0 points; high expression, 40 points) in the ICGC cohort, and higher total points were associated with a poor prognosis. The calibration curves indicated a clear consistency between the predicted and actual clinical survival outcomes. Additionally, C‐index and AUC value for the TCGA and ICGC cohorts were 0.6361 (95% CI = 0.5818–0.6904) and 0.7470 (95% CI = 0.6780–0.8159), respectively. In brief, the predictive mold can be relied on to predict the prognosis of HCC patients, and PSMB5 expression indicated a stable predictive ability.

**Table 3:** Independent prognostic factor analysis for the overall survival in HCC based on TCGA cohort.

Characteristics	Univariate analysis				Multivariate analysis			
	HR	HR.95L	HR.95H	*P* value	HR	HR.95L	HR.95H	*P* value
Gender (female vs. male)	0.776	0.531	1.132	0.188	–	–	–	–
Age (≥ 65 years vs. < 65 years)	1.229	0.847	1.782	0.278	–	–	–	–
Grade (1 vs. 2 vs. 3 vs. 4)	1.133	0.881	1.457	0.330	–	–	–	–
Clinical stage (I vs. II vs. III vs. IV)	1.680	1.369	2.062	< 0.0001	1.669	1.358	2.051	< 0.0001
PSMB5 expression (high vs. low)	1.610	1.109	2.337	0.0123	1.553	1.069	2.258	0.021

**Table 4:** Independent prognostic factor analysis for the overall survival in HCC based on ICGC cohort.

Characteristics	Univariate analysis				Multivariate analysis			
	HR	HR.95L	HR.95H	*P* value	HR	HR.95L	HR.95H	*P* value
Gender (female vs. male)	0.519	0.278	0.966	0.037	0.416	0.220	0.787	0.007
Age (≥ 65 years vs. < 65 years)	0.820	0.404	1.665	0.583	–	–	–	–
Clinical stage (I vs. II vs. III vs. IV)	2.155	1.493	3.110	< 0.0001	0.416	0.220	0.787	0.007
Prior malignancy	1.751	0.773	3.965	0.179	–	–	–	–
PSMB5 expression (high vs. low)	3.013	1.544	5.882	0.001	2.509	1.271	4.950	0.008

### Genetic alteration analysis of PSMB5

**Fig. 6:**
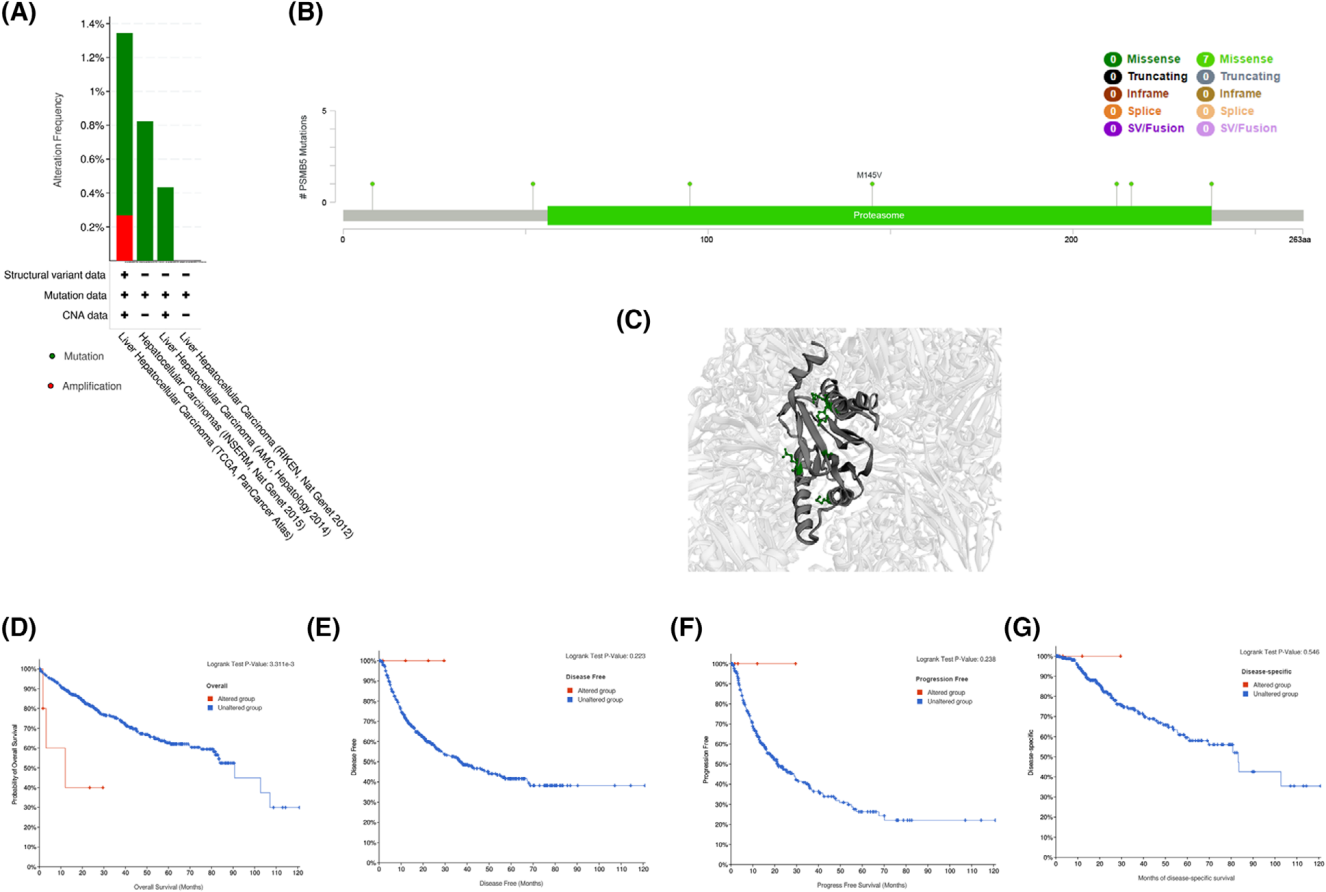
Genetic alteration of PSMB5 in HCC. (A) The alteration frequency with mutation type of PSMB5. (B) Mutation sites of PSMB5. (C) The mutation site in the 3D structure of PSMB5. (D–G) Survival analysis of overall survival, disease‐free survival, progression‐free survival and disease‐specific survival with or without PSMB5 alteration.

The cBioPortal tool was used to investigate the mutation features of PSMB5 in HCC. We found that the genetic alteration frequency of PSMB5 ranged from 0.43% to 1.34%, with a missense mutation type of genetic alteration (Fig. 6A,B). Mutation sites of PSMB5 and its 3D structure in LIHC are shown in (Fig. 6C). LIHC cases with altered PSMB5 showed a poor prognosis in OS (*P* = 0.0033), whereas there was similar disease‐specific, disease‐free (*P* = 0.223) and progression‐free (*P* = 0.546) survival compared to cases without PSMB5 alteration (Fig. 6D–G).

### Tumor immune infiltration of PSMB5

**Fig. 7:**
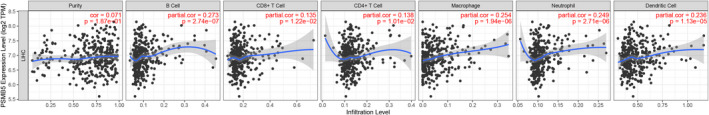
Association of PSMB5 expression with immune infltration levels in HCC from the TCGA.

The correlation between PSMB5 expression and immune infiltration in HCC was explored by using TIMER database. According to the results, PSMB5 expression was positively correlated with B cells, CD4^+^ T cells, CD8^+^ T cells, dendritic cell, macrophages and neutrophils. However, PSMB5 expression was not significantly associated with tumor purity (Fig. 7). Moreover, PSMB5 expression was positively correlated with the immune marker genes of immune cells, such as NRP1, HLA‐DPA1, HLA‐DRA, HLA‐DPB1, IRF5, CSF1R, CD86, ITGAM, HAVCR2, CD68, BCL6, STAT1, STAT5A and TGFB1 (Table 5). The relationship between PSMB5 expression and the above‐mentioned markers genes was further validated in the GEPIA database. These findings suggest that the high level of PSMB5 expression was closely associated with immunity in HCC.

**Table 5:** Association analysis between PSMB5 and immune cells markers base on TIMER and GEPIA database.

Cell type	Gene marker	TIMER				GEPIA			
		None		Puilty		Tumour		Normal	
		Cor	*P*	Cor	*P*	Cor	*P*	Cor	*P*
B cell	CD19	0.123	*	0.146	**	0.035	0.51	0.18	0.21
	CD79A	0.022	0.677	0.06	0.263	0.023	0.65	0.29	*
CD8^+^ T cell	CD8A	0.046	***	0.086	0.112	0.063	0.23	0.39	**
	CD8B	0.043	0.405	0.081	0.133	0.15	**	0.4	**
Dendritic cell	ITGAX	0.146	**	0.2	***	0.071	0.18	0.15	0.29
	NRP1	0.262	***	0.267	***	0.24	***	0.17	0.23
	CD1C	0.047	0.366	0.058	0.281	0.092	0.077	0.36	**
Natural killer cell	HLA‐DPA1	0.109	*	0.142	**	0.15	**	0.24	*
	HLA‐DRA	0.127	*	0.165	*	0.16	**	0.31	*
	HLA‐DQB1	0.105	*	0.138	*	0.069	0.19	0.19	0.17
	HLA‐DPB1	0.134	*	0.168	**	0.17	***	0.34	*
	KIR2DS4	0.032	0.535	0.023	0.674	0.094	0.071	0.094	0.52
	KIR3DL3	0.01	0.842	0.004	0.934	0.04	0.44	0.32	*
	KIR3DL2	0.039	0.459	0.06	0.269	0.05	0.34	0.079	0.58
	KIR2DL4	0.041	0.427	0.064	0.233	0.071	0.17	0.33	*
	KIR2DL3	0.085	0.101	0.102	0.059	0.057	0.28	0.26	0.064
	KIR2DL1	0.017	0.740	0.006	0.914	0.073	0.16	0.38	**
M1 macrophage	PTGS2	0.037	0.480	0.062	0.252	−0.059	0.26	0.24	0.097
	IRF5	0.209	***	0.21	***	0.14	**	0.34	*
	NOS2	0.015	0.774	0.013	0.814	−0.058	0.26	0.28	*
M2 macrophage	MS4A4A	0.067	0.198	0.108	*	0.1	*	0.36	*
	VSIG4	0.043	0.412	0.067	0.215	0.14	**	0.29	*
	CD163	0.046	0.372	0.068	0.211	0.11	*	0.26	0.069
Monocyte	CSF1R	0.145	***	0.184	***	0.17	**	0.32	*
	CD86	0.168	**	0.222	***	0.17	***	0.3	*
Neutrophils	CCR7	0.012	0.813	0.039	0.471	0.0083	0.87	0.33	*
	ITGAM	0.182	***	0.196	***	0.13	**	0.43	**
	CEACAM8	0.025	0.636	0.024	0.657	−0.035	0.5	0.36	*
T cell (general)	CD3D	0.066	0.283	0.102	0.059	0.072	0.17	0.37	**
	CD3E	0.044	0.899	0.088	0.145	0.052	0.32	0.28	*
	CD2	0.038	0.471	0.079	0.145	0.059	0.26	0.2	0.17
T cell exhaustion	LAG3	0.039	0.457	0.064	0.239	0.13	*	0.28	*
	HAVCR2	0.157	**	0.214	***	0.17	**	0.25	0.075
	GZMB	0.057	0.277	0.08	0.138	0.13	*	0.51	***
	PDCD1	0.112	*	0.156	**	0.063	0.23	0.42	**
TAM	CCL2	0.014	0.785	0.018	0.733	0.14	**	0.27	0.056
	IL10	0.139	*	0.177	***	0.1	0.054	0.5	**
	CD68	0.207	***	0.254	***	0.14	**	0.35	*
Tfh	BCL6	0.213	***	0.211	***	0.15	**	−0.1	0.47
	IL21	0.068	0.194	0.096	0.075	0.0081	0.88	0.21	0.15
Th1	TBX21	0.003	0.953	0.021	0.613	0.024	0.65	0.4	**
	STAT4	0.018	0.725	0.012	0.830	0.11	*	0.3	**
	STAT1	0.228	***	0.25	***	0.19	***	0.59	***
	IFNG	0.1	0.054	0.142	**	0.084	0.11	0.29	*
	IL13	0.027	0.601	0.046	0.393	0.053	0.31	0.063	0.66
Th2	GATA3	0.051	0.332	0.088	0.103	0.13	*	0.066	0.65
	STAT6	0.166	*	0.145	**	0.077	0.14	0.52	***
	STAT5A	0.249	***	0.254	***	0.15	**	0.51	**
Th17	STAT3	0.102	*	0.108	*	0.052	0.32	−0.09	0.53
	IL17A	0.015	0.770	0.023	0.671	0.066	0.21	0.14	0.32
Treg	FOXP3	0.007	0.894	0.016	0.768	−0.21	*	0.35	*
	CCR8	0.163	**	0.197	***	0.086	0.099	0.15	0.3
	TGFB1	0.252	***	0.296	***	0.25	***	0.39	**

* *P* < 0.05

** *P* < 0.01

*** *P* < 0.001.

### PSMB5 regulated cell proliferation, apoptosis and migration *in vitro*

**Fig. 8:**
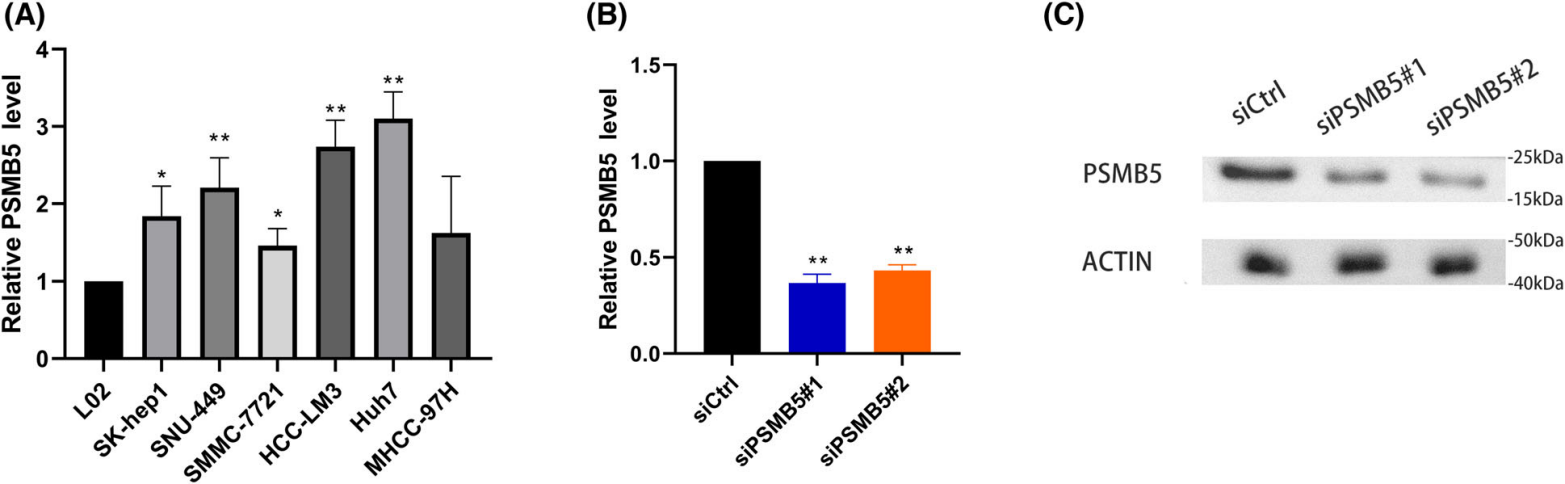
PSMB5 expression level in HCC cell lines and construction of HCC cell models with PSMB5 knockdown. (A) Levels of psmb5 mRNA in difference HCC cell lines. (B, C) qPCR and western blotting were applied to examine the knockdown efficiency of PSMB5 in huh7. Unpaired two‐tailed Student's *t*‐tests were used to determine significance. Error bars indicate the SD. The precise *n* value (number of biologically‐independent replicates) is 3, except for western blotting where *n* = 1. *P < 0.05 **P < 0.01.

**Fig. 9:**
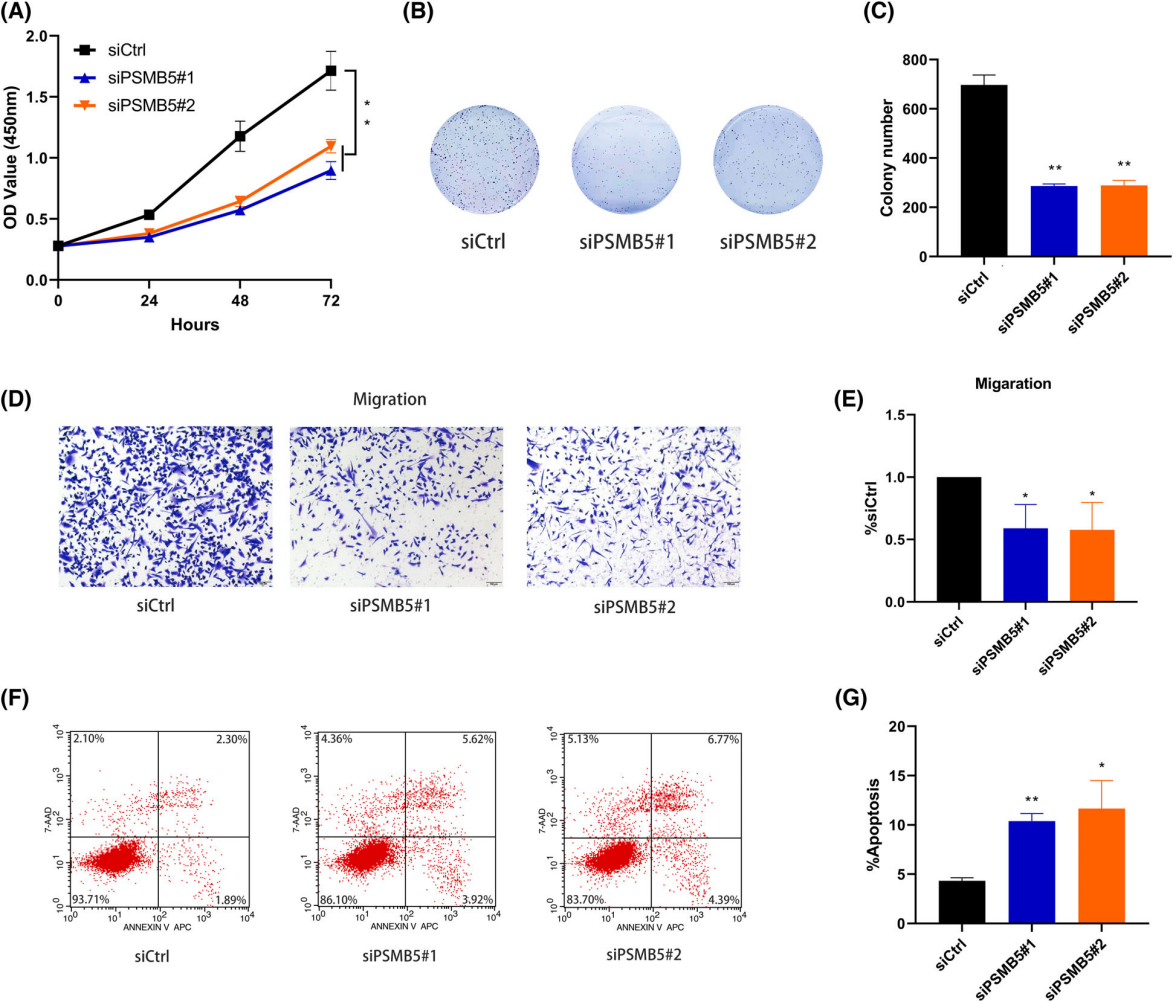
PSMB5 modulated proliferation, migration and apoptosis *in vitro*. (A) A CCK‐8 assay was conducted to detect the proliferation of PSMB5 knockdown in huh7. (B, C) The effect of PSMB5 knockdown on the colony forming capacity of huh7. (D, E) The effect of PSMB5 knockdown on the migration capacity of huh7. Scale bars = 100 μm. (F, G) The effect of PSMB5 knockdown on the cell apoptosis in huh7. Unpaired two‐tailed Student's *t*‐tests and one‐way or two‐way analysis of variance were used to determine significance. Error bars indicate the SD. The precise *n* value (number of biologically‐independent replicates) is 3. *P < 0.05 **P < 0.01.

The results of a qRT‐PCR assay showed that PSMB5 expression was significantly increased in HCC cell lines (SK‐hep1, SNU‐449, SMMC‐7721, HCC‐LM3 and Huh7) compared to normal liver cell line L02 (Fig. 8A). Considering the highest level of PSMB5 expression in huh7, huh7 was selected to further explore the biological function of PSMB5 in HCC. After siRNA targeting PSMB5 was transfected into huh7 cells, the level of PSMB5 expression in the siRNA1 and siRNA2 groups was significantly reduced relative to the negative control group (Fig. 8B,C). According to the result of CCK‐8 assays and colony formation, the suppression of PSMB5 expression could significantly reduce the proliferation ability of HCC cells (Fig. 9A–C). The knockdown of PSMB5 significantly reduced the migratory ability of HCC cells (Fig. 9D,E). Meanwhile, the downregulation of PSMB5 expression could significantly enhance apoptosis (Fig. 9F,G).

### PSMB5 inactivates the PI3K/Akt/mTOR signaling pathway in huh7

**Fig. 10:**
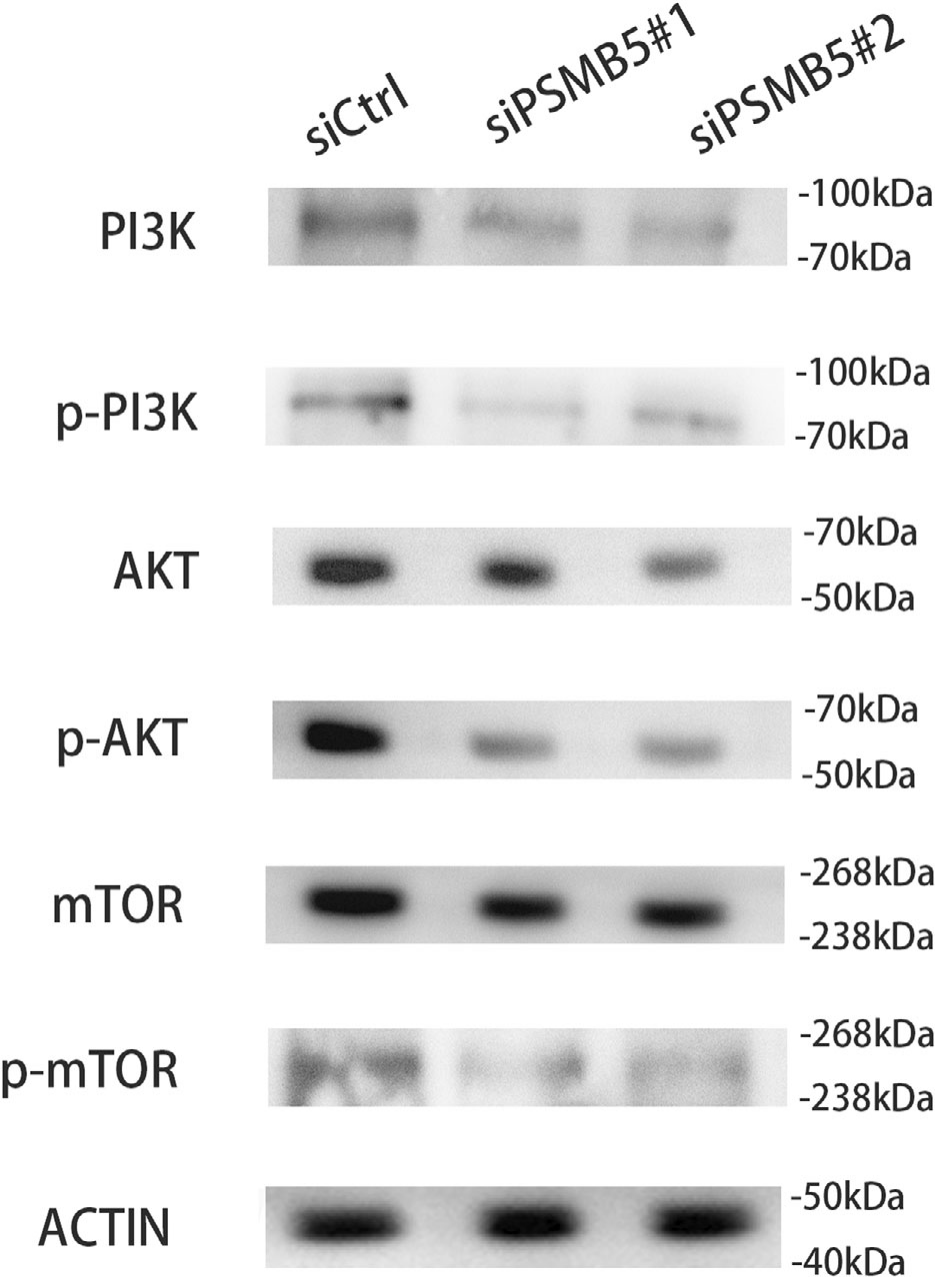
PSMB5 regulates the PI3K/Akt/mTOR signaling pathway. Western blotting was performed against the indicated proteins in huh7 cells treated with siCtrl siPSMB5#1 or siPSMB5#2.

It is established that the PI3K/Akt/mTOR signaling pathway is implicated in HCC progression [15, 16, 17]. Thus, we examined whether PI3K/Akt/mTOR signaling could be modulated by PSMB5. The results indicated that the levels of p‐PI3K, p‐Akt and p‐mTOR in slicing groups were significantly reduced compared to the control group (Fig. 10). In summary, slicing PSMB5 may suppress the proliferation of HCC cells through inhibition of the PI3K/Akt/mTOR signaling pathway.

### PSMB5 affects the 20S proteasome chymostrypsin‐like activity

**Fig. 11:**
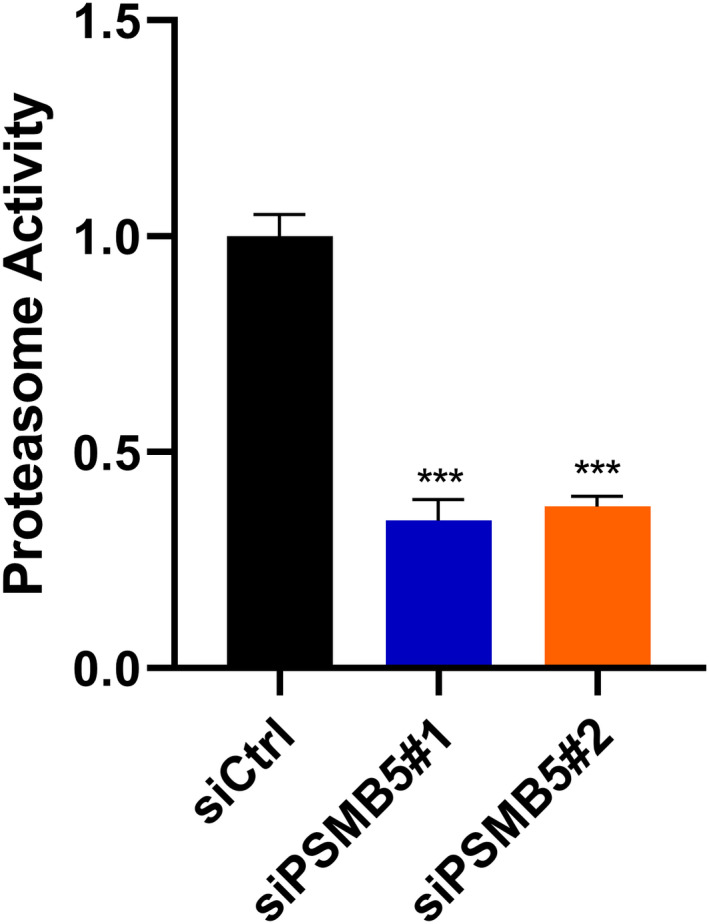
Proteasome activity of huh7 cells treated with siCtrl, siPSMB5#1 or siPSMB5#2 were detected using a Proteasome 20S Activity Assay Kit (Abcam) with a fluorescence microplate reader. Unpaired two‐tailed Student's *t*‐tests were used to determine significance. Error bars indicate the SD. The precise *n* value (number of biologically‐independent replicates) is 3. ***P < 0.001.

The chymotrpsin‐like activity of the 20S proteasome activity in hu7 cells was assessed, and the results indicated that the inhibition of PSMB5 significantly reduced hu7 cell chymotrpsin‐like activity (Fig. 11).

## Discussion

HCC, with its rising incidence, remains a tough challenge for public health worldwide [18]. As a result of the complex biological mechanism of HCC and the heterogeneity of cancer cells, the survival rate of HCC patients remains low [19]. Therefore, identifying new biomarkers for HCC plays a critical role in enabling early disease detection and improving prognostic value for HCC patients. The occurrence of HCC involves many types of gene mutations, thus leading to an increase of protein synthesis [20]. Compared to normal cells, the proteasome level and activity are higher in HCC cells [21]. Some members of the PSMB family, such as PSMB2, PSMB4, PSMB7 and PSMB8, have been demonstrated to be associated with HCC proliferation, invasion and drug resistance [22, 23, 24, 25]. PSMBs subunits are also closely associated with tumorigenesis. As one component of the active center of the 20S proteasome, PSMB5 plays a critical role in regulating the content of particular proteins in the human body and in removing misfolded proteins [26]. PSMB5 overexpression could enhance the capability of human lens epithelial cells to resist oxidative stress, enhance the viability of cells and reduce the content of oxidized protein [27]. Currently, there is still little research on the expression and function of PSMB5 in HCC. In the present study, the bioinformatics method revealed that PSMB5 was overexpressed in HCC tissues compared to the normal tissues. A qRT‐PCR and IHC validated the results. The high level of PSMB5 expression was associated with worse overall survival. A predictive mold was built based on clinical factors and the level of PSMB5 expression. Furthermore, it was observed that the knockdown of PSMB5 expression reduced the proliferation and migration of HCC cells but increased apoptosis. A flow chart is shown in Fig. S1.

Because of the important role of PSMB5 in mediating proteasome function, the abnormal expression of PSMB5 reflected the dysfunction of the whole proteasome complex in cancer. The proteasome activity of PSMB5 has been demonstrated to be induced by STAT3‐related oncogenic signaling [28]. Wei *et al*. [11] demonstrated that the expression of PSMB5 in triple‐negative breast cancer (TNBC) was significantly higher than in normal tissues, the high level of PSMB5 expression was correlated with worse OS and progression free survival, and PSMB5 knockdown promoted the apoptosis of TNBC cells and sensitized TNBC cell to chemotherapeutic agents. As reported by Fan *et al*. [12], the downregulation of miR‐127‐3p enhanced the cell invasion and migration of prostate cancer *in vitro* by upregulating the PSMB5. Wang *et al*. [7] revealed that the downexpression of PSMB5 significantly inhibited the proliferation and migration of breast cancer cells both *in vitro* and *in vivo*. Fang *et al*. [13] demonstrated that hsa_circ_0000700 could activate PSMB5 indirectly to enhance esophageal squamous cell carcinoma proliferation. These findings suggest an oncogenic role of PSMB5, which is consistent with our research results.

As a proteasome inhibitor, bortezomib has been widely used for anticancer therapy, and PSMB5 is the target of bortezomib [29, 30, 31]. Furthermore, some reports have indicated that the mutation or upregulation of PSMB5 protein contributed to bortezomib drug‐resistance [32, 33, 34, 35]. In the present study, more genetic alterations to PSMB5 were linked to a poor OS for HCC patients, indicating that the genetic alterations to PSMB5 might play an important role in HCC.

The immune cells in the tumor microenvironment are essential for tumorigenesis, and many immune cells may function as a tumor promoter or inhibitor in HCC [36]. The occurrence and development of HCC are accompanied by immune resistance and immune evasion [37]. Our results showed that PSMB5 expression was significantly correlated with the infltration levels of B cells, CD4^+^ T cells, CD8^+^ T cells, dendritic cell, macrophages and neutrophils. Macrophages are involved in the immune responses to tumors in a polarized manner ^38^. M1 macrophages contribute to tumoricidal responses by producing interleukin‐12, whereas M2 macrophages contribute to tumor progression by producing interleukin‐10 [38, 39]. In the study by Wang *et al*. [7], the inhibition of PSMB5 in immune cells could promote potent anti‐tumor immunity by enhancing M1 macrophage activity and reducing the number of M2 macrophages. One study suggested that macrophages prevent CD8 T cells from reaching tumor cells and thus weaken the effectiveness of anti‐PD‐1 therapies [40]. These findings reveal the potential regulatory role of PSMB5 in tumor‐associated macrophages. Moreover, the association between PSMB5 expression and the genes sets of immune cells implied a critical role of PSMB5 in regulating tumor immunology in HCC. It was discovered that the gene markers of monocyte (CSF1R and CD86) were positively associated with PSMB5 expression. A previous study showed that the chemokines from monocyte and the signals from tumor microenvironment contributed to the production of the pro‐metastatic factor oncostatin M by neutrophils, which may be potential targets of immune‐based anticancer treatment for HCC patients [41]. A lower amount of monocytes was correlated with a significantly higher OS in HCC [42]. In addition, PSMB5 expression was significantly associated with the regulation of several immune genes, such as CD68, HAVCR2 and NRP1. As a type of myeloid‐specific surface marker, CD68 is expressed by macrophages at fairly high levels [43]. Also known as Tim‐3, HAVCR2 is considered as an immune checkpoint molecule and plays complex and important roles in mediating immune responses and tolerance [44]. Anti‐Tim‐3 antibodies have an impact on the phenotype of myeloid cells in the tumor microenvironment [45]. A larger amount of Tim‐3^+^ tumor‐infiltrating T cells in HCC was associated with the worse survival outcome [46]. NRP1 could promote many processes of tumorigenesis, including angiogenesis, apoptosis, migration, invasion and drug resistance [47]. Currently, NRP1 is known as a critical barrier for anti‐tumor immunity [48]. The study of Lin revealed that NRP1 was overexpressed in HCC tissues and cell lines, and that the inhibition of NRP1 suppressed the transformative phenotypes in HCC cells [49].

As an important intracellular signal transduction pathway, the PI3K/Akt/mTOR pathway is involved in tumor development, cellular metastasis and proliferation. A previous study indicated that the PI3K/Akt/mTOR pathway was overexpressed in 40–50% of HCC samples [15]. In the present study, it was found out that the downregulation of PSMB5 significantly reduced the phosphorylation level of proteins for the PI3K/Akt/mTOR signaling pathway. A study by Deng *et al*. [16] showed that the overexpression of XPA not only inhibits proliferation, migration and invasion in HCC, but also suppresses the expression of p‐PI3K, p‐Akt and p‐mTOR. Luo *et al*. [17] revealed that YTHDF1 promoted the proliferation of HCC cells by activating the PI3K/Akt/mTOR signaling pathway. Wang *et al*. [50] demonstrated that MiR‐92a‐3p increased the malignant progression of HCC by activating the phosphorylation level of proteins associated with the PI3K/Akt/mTOR signaling pathway. These findings were essentially consistent with ours.

As far as we know, the present study is the first to explore the potential role of PSMB5 in HCC, despite some limitations. On the one hand, the effect of PSMB5 in HCC should be validated *in vivo*. On the other hand, our findings must be verified using more clinical samples.

## Conclusions

In summary, PSMB5 might serve as a potential marker for the molecular diagnosis and prognosis of HCC. Moreover, PSMB5 knockdown inhibited the proliferation and migration of HCC cells at the same time as increasing apoptosis, which may be regulated by PI3K/Akt/mTOR signaling. These findings might offer new clues to understanding the molecular mechanisms of PSMB5 in HCC.

## Conflicts of interest

The authors declare that they have no conflicts of interest.

## Author contributions

JL and LJ conceived and designed the project, JM acquired the data, SL and HC analysed and interpreted the data, JL wrote the paper.

## Supporting information


**Fig. S1.** Flow chart of the present study.

**Table S1.** The top 10 relevant genes of KIF15.

## Data Availability

The datasets generated in the present study are available from the corresponding author upon reasonable request.
