# Supplementary figures and images for: PSMB5 overexpression is correlated with tumor proliferation and poor prognosis in hepatocellular carcinoma
Source: FEBS Open Bio. 2022 Sep 22;12(11):2025–41. doi: 10.1002/2211-5463.13479 (PMC9623531; doi:10.1002/2211-5463.13479)

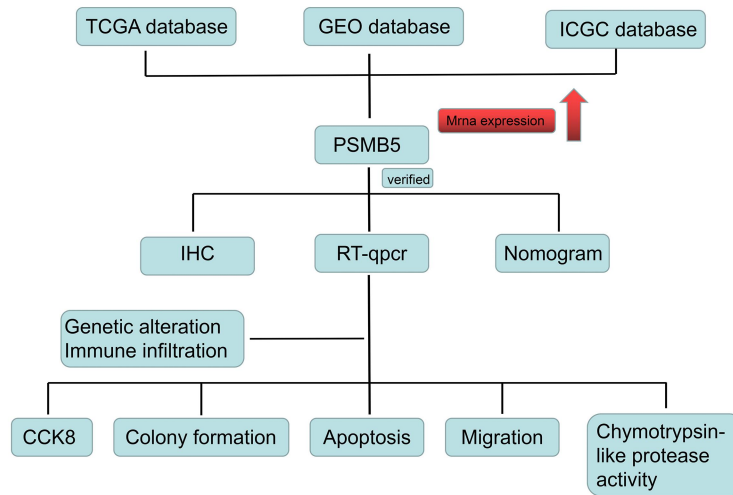

Supplementary Figure 1. Flow chart of the present study.

Supplement: Supplementary file 1 — Fig. S1. Flow chart of the present study. [file FEB4-12-2025-s001.pdf]
