# Supplementary material for: PSMB5 overexpression is correlated with tumor proliferation and poor prognosis in hepatocellular carcinoma
Source: FEBS Open Bio. 2022 Sep 22;12(11):2025–41. doi: 10.1002/2211-5463.13479 (PMC9623531; doi:10.1002/2211-5463.13479)
Supplement: Supplementary file 2 — Table S1. The top 10 relevant genes of KIF15. [file FEB4-12-2025-s002.doc]

Supplementary table1. The top 10 relevant genes of KIF15.

| Gene Symbol | Gene ID | PCC |
| --- | --- | --- |
| NEDD8 | ENSG00000129559.12 | 0.72 |
| OXA1L | ENSG00000155463.12 | 0.63 |
| DAD1 | ENSG00000129562.10 | 0.62 |
| APEX1 | ENSG00000100823.11 | 0.62 |
| PRMT5 | ENSG00000100462.15 | 0.61 |
| IPO4 | ENSG00000196497.15 | 0.6 |
| C14orf119 | ENSG00000179933.5 | 0.59 |
| CHD8 | ENSG00000100888.12 | 0.58 |
| NGDN | ENSG00000129460.15 | 0.57 |
| CCT7 | ENSG00000135624.15 | 0.56 |

PCC, Pearson correlation coefficient.
